# A randomized controlled trial to evaluate the progressive muscle relaxation technique in hip fracture patients

**DOI:** 10.1038/s41598-024-64516-4

**Published:** 2024-06-12

**Authors:** Sahar Mashhadi-Naser, Saeid Shirvani, Parvaneh Vasli

**Affiliations:** 1 Student Research Committee, Department of Community Health Nursing, School of Nursing and Midwifery, Shahid Beheshti University of Medical Sciences, Tehran, Iran; 2 Department of Orthopedics, Orthopedic Research Center, School of Medical Sciences, Shohadaye Haftome Tir Hospital, Iran University of Medical Scinences, Tehran, Iran; 3 Department of Community Health Nursing, School of Nursing and Midwifery, Shahid Beheshti University of Medical Sciences, Vali Asr Ave., Ayatollah Hashemi Rafsanjani Cross Road, Tehran, Iran

**Keywords:** Relaxation, Hip fracture, Pain, Sleep quality, Anxiety, Neuroscience, Psychology, Health care, Medical research

## Abstract

The purpose of this research was to ascertain how progressive muscle relaxation (PMR) technique affected hip fracture patients' anxiety, sleep quality, and post-operative pain. This parallel randomized controlled trial was conducted on 100 patients with hip fracture hospitalized in one of the reference orthopedic hospitals in Tehran, Iran who were selected using convenience sampling and randomly were placed in two PMR group (n = 50) and control group (n = 50). Data were collected by Demographic information questionnaire, Visual analogue scale for pain rating, Pittsburgh Sleep Quality Index and State-Trait Anxiety Inventory. The PMR technique was the progressive muscle relaxation technique, which was started the night after the surgery for three nights before going to bed. Data were collected on two occasions, including before the PMR technique and the day after the last stage of the PMR technique. The data were analyzed by SPSS software using descriptive and inferential statistics. The results revealed significant within-group changes in both groups' post-operative pain, sleep quality, and anxiety scores (*P* < 0.001). The progressive muscle relaxation group experienced decreased post-operative pain and anxiety scores and increased sleep quality scores (*P* < 0.001). The linear mixed model showed that the absolute changes in the follow-up post-operative pain, sleep quality, and anxiety scores were 1.19 and 7.94 units, significantly lower than the baseline, respectively. The results revealed significant within-group changes in both groups' post-operative pain, sleep quality, and anxiety scores (*P* < 0.001). The progressive muscle relaxation group experienced decreased post-operative pain and anxiety scores and increased sleep quality scores (*P* < 0.001). The study's findings demonstrated the beneficial effects of progressive muscle relaxation on hip fracture patients' outcomes, such as their level of anxiety, sleep quality, and post-operative pain. The study's findings can be applied by medical professionals to improve patient satisfaction and care quality.

This clinical trial has been registered with the Iranian Registry of Clinical Trials under the code IRCT20231120060119N1, which was approved on 7/12/2023.

## Introduction

According to the literature, femur and hip fractures (HF) are the most common orthopedic fractures, especially in the elderly population^1^. Iran's population is ageing rapidly due to rapid demographic changes in the past, in particular a sharp decline in fertility and an increase in life expectancy^2^.

HF occurs in older people following mild trauma^1^. In the majority of cases of HF, early surgery is performed. One of the most common and debilitating complications after surgery is pain^3,4^. The consequences of uncontrolled acute postoperative pain (POP) can be long-term and negative. These consequences can manifest six months later and include increased mortality rates, pain that persists, reduced ambulation, and a reduced ability to return to community life^5^.

A substantial body of evidence indicates that sleep quality (SQ) and pain are interrelated. Pain, in turn, is a common side effect of sleep disturbance, and vice versa^6^. SQ is defined as a person's overall satisfaction with their sleep experience, encompassing four attributes: sleep efficiency, sleep latency, sleep duration, and wake after sleep onset^7^. It is possible that fractures such as HF may result in sleep disturbances. The recovery from a painful HF and surgery may result in sleep disturbances in some cases^8^.

In addition to experiencing pain and disturbed sleep, older adults with HF may also encounter a multitude of anxiety-provoking scenarios, including those involving unfamiliar or unpleasant situations, such as traveling by ambulance to reach the hospital, facing an emergency or needing surgery, and leaving the operating room under the effects of anesthesia without being adequately informed^9^. Furthermore, it has been demonstrated that POP can cause anxiety in patients who have undergone hip surgery. Anxiety can be classified as trait anxiety and state anxiety. Trait anxiety is defined as a person's sensitivity to anxiety, whereas state anxiety is understood to be a temporary state of tension or worry caused by a potential threat. Anxiety related to surgery is considered a state anxiety^9^.

In addition to the importance of reducing SQ and anxiety as variables affecting health, pain as a complication of HF requires management. Various methods are used for pain management, including intravenous administration of non-steroidal drugs, opiates, nerve blocks, and so forth^10^. However, non-drug methods are safe and without side effects in most cases. A significant proportion of these methods are included within the domain of complementary medicine^11^.

Progressive muscle relaxation (PMR) is the most readily accessible and prescribed technique for inducing physical and mental relaxation^12^. This technique is associated with breathing exercises and includes the tension and relaxation of different groups of skeletal muscles^13^. The relaxation that occurs during PMR has been shown to reduce anxiety, release muscle tension, regulate neuromuscular activity, and reduce various forms of pain sensation by blocking the transmission of pain stimuli and changing pain perception^12^. The technique in question is relatively simple to learn and can be employed to relieve a number of symptoms, including distraction, tension, muscle contractions, stress and anxiety, sleep disturbances and sensitivity to pain and fatigue. Furthermore, PMR does not necessitate a specific time or place, nor does it require any special technology or equipment^11^.

A number of recent studies have investigated the impact of PMR on patients' pain, anxiety, and SQ. In one of the studies, Chen et al.^14^ used the difference-in-differences model to assess the impact of PMR on stress, anxiety, and POP in patients who had lumbar disc herniation. Another study looked into how PMR affected patients with head and neck cancer's vital signs, POP, and level of fatigue^15^. Eymir et al.^12^ compared the pain relief, function and neuromuscular outcomes of the simultaneous use of PMR and standard physiotherapy compared to the use of only standard physiotherapy in patients undergoing total knee arthroplasty. In Italy, De Paolis et al.^16^ studied the effect of interactive guided imagery and PMR on pain relief in hospice patients at the end stage of cancer. Another study was conducted in Iran with the aim of investigating the effect of Benson relaxation and PMR techniques on SQ of patients undergoing coronary artery bypass graft surgery^17^. Masry et al.^18^ investigated the effect of Benson's relaxation technique on night pain and SQ in adults and elderly patients undergoing joint replacement surgery in Egypt. A study aimed at investigating the feasibility, acceptance and initial effects of music intervention with PMR on anxiety, depression, stress and quality of life among breast cancer patients and women undergoing chemotherapy^19^. In another study in southern China, the effect of PMR on state anxiety and self-efficacy of patients hospitalized due to limb fracture undergoing elective surgery was investigated^20^.The aim of the study was to investigate the effect of PMR on POP, SQ, and anxiety in a cohort of Iranian HF patients (HFPs), for whom no previous research had been conducted on this topic. Accordingly, the study proposed three hypotheses for the study as follows:

- H1: The PMR technique is effective at the POP level of HFPs.
- H2: The PMR technique is effective at the SQ level of HFPs.
- H3: The PMR technique is effective at the anxiety level of HFPs.

## Methods

### Design and setting

The present research is a parallel randomized controlled trial conducted at the 7th Tir Martyrs Hospital in Tehran, Iran. This hospital is a specialist center for orthopedic and trauma care, with three dedicated orthopedic departments comprising 40 beds. Two of these are for male patients and one is for female patients. It is important to note that, in accordance with Iranian legislation, women and men must be treated in separate departments.

This clinical trial has been registered with the Iranian Registry of Clinical Trials under the code IRCT20231120060119N1, which was approved on 7/12/2023.

### Participants and recruitment

The participants of the study were HFPs who were hospitalized in the orthopedic departments of 7th Tir Martyrs Hospital. The sample size based on the study of Masry et al.^18^ considering α = 0.05, β = 0.10, power of the study equal to 0.90, and 20% dropout rate and using the following formula was estimated equal to 50 people in each of control group (CG) and PMR group (PMRG).

$$n \ge 2\frac{{(z_{\alpha /2} + z_{\beta } )^{2} \sigma^{2} }}{{(\mu_{1} - \mu_{2} )^{2} }}$$

The inclusion criteria for the participants to enter the study were: confirmed diagnosis of HF, age 40 to 65 years, not to suffer from multiple trauma or fractures of both upper and lower limbs, having reading and writing literacy, not taking sleeping, sedative and anti-depressant or psychotic drugs in the past month, not suffering from dementia or serious cognitive dysfunction based on the self-report of the patient or his family, lack of drug addiction according to the information included in the file and not receiving narcotic drugs after surgery. The exclusion criteria were the patient's death, the patient's refusal to continue participating in the study, and the need for re-surgery.

The first and second authors were responsible for the selection of participants and their subsequent allocation to the groups. Sampling continued for a period of three months until the estimated sample size was achieved. The initial cohort comprised 155 HFPs aged between 40 and 65 years, who had been hospitalized for surgery and did not have multiple traumas. A total of 25 HFPs were excluded from the study due to a lack of other study criteria, including reading and writing literacy, drug addiction, cognitive dysfunction, or the use of drugs related to psychotic disorders. A total of 130 eligible participants were randomly allocated to the PMRG (n = 65) and the CG (n = 65) using a coin and toss method. Following the implementation of the intervention in the PMRG, 20 participants from both groups were excluded due to deterioration and transfer to the ICU, personal consent before discharge by the attending physician, and death. Furthermore, 10 participants from both groups were excluded due to their refusal to continue participating in the study. Finally, the data from the 50 participants in the PMRG and the 50 participants in the CG were subjected to analysis. It is notable that the participants in both groups were comparable in terms of the type and timing of their receipt of analgesics following surgery. It is crucial to highlight that the participants in both groups were comparable in terms of the type and timing of their receipt of analgesics following surgery. Following consultation with the medical team, which was conducted by the second researcher, all participants in the two groups were administered Apotel three times a day (every eight hours) following surgery and until the conclusion of the intervention. In accordance with the established inclusion criteria, which encompassed the absence of multiple trauma or fractures of both upper and lower limbs, the use of sleeping, sedative, and anti-depressant or psychotic drugs in the preceding month, the absence of drug addiction as indicated in the file, and the absence of narcotic drug use following surgery, Apotel was deemed an adequate analgesic for all participants.

### Outcome measurements

#### Baseline

Baseline data were collected by the first researcher. The data was collected prior to the implementation of the PMRG, which occurred the night after surgery. For those participants who were illiterate, the questions were read in a monotone voice and answered by the first researcher.

#### Follow-up

**Figure 1:**
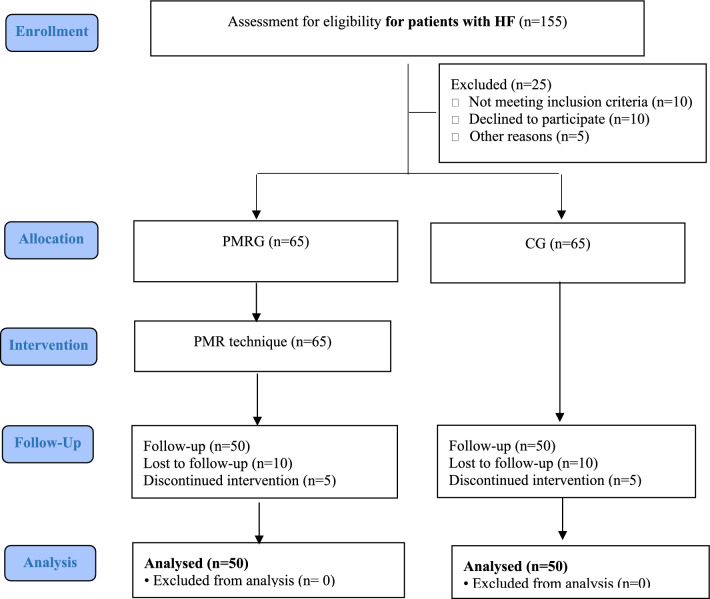
CONSORT flow chart of the study.

The data pertaining to the follow-up phase was collected by the first researcher. The measurement was conducted on the fourth day following the surgical procedure, which was the day after the conclusion of the PMRG. The study flowchart is presented in Fig. 1.

### Measures

#### Demographic information questionnaire

The questionnaire comprised eight questions pertaining to the following variables: age, gender, marital status, education level, occupation, the initial role within the family, the number of family members, and the health insurance status, and injury mechanism.

#### Visual analogue scale for pain rating

In this study, the Visual Analogue Scale for Pain Rating was employed to quantify the severity of POP. This scale represents a valid method for measuring mental states associated with acute and chronic pain. The patient is typically invited to indicate the intensity of their pain on a scale ranging from zero (no pain) to 10 (the worst imaginable pain), or to indicate their pain by means of a circle around the number. The scale's advantages include simplicity, reproducibility, ease of comprehension and sensitivity to minor fluctuations in pain^21^. The validity and reliability of the Pain Rating Scale have been corroborated in a multitude of studies^22^.

#### The Pittsburgh *SQ Index*

The Pittsburgh SQ Index was developed and validated in 1989 by Buysse et al. This self-report instrument is designed to assess SQ and its disturbances over the course of a month. The tool consists of 19 items and seven components, including subjective sleep quality, sleep latency, sleep duration, normal sleep efficiency, sleep disturbances, use of sleeping pills or medication, and impairment of daytime functioning (daytime dysfunction). The sum of the scores for the seven components gives a total score^23^. Responses are scored on a scale of 0 to 3, with a score of 3 reflecting extreme negativity on the Likert scale. The maximum score is 21 and a score of 5 or more indicates poor sleep^24^.

The Pittsburgh SQ Index has a Cronbach's alpha coefficient of 0.83. In numerous studies that have employed the Pittsburgh SQ Index in diverse elderly populations across the globe, its validity and reliability have been substantiated^24^. In Iran, this tool has been validated for 123 patients with psychiatric disorders and 133 healthy individuals, with a Cronbach's alpha of 0.77 obtained for the entire instrument^22^. In this study, the Cronbach's alpha coefficient was 0.91.

#### The State-Trait Anxiety Inventory

The State-Trait Anxiety Inventory was employed as a measurement tool, comprising 40 statements divided into two sub-scales: the State Anxiety Index and the Trait Anxiety Index. In this study, the State Anxiety Index section, comprising statements 1 to 20, was employed. The responses were provided on a 4-point Likert scale ranging from "not at all" (score 1) to "very much" (score 4). A higher total score indicates more anxiety. The scoring of the ten statements included in the items numbered 20, 19, 16, 15, 11, 10, 8, 5, 2, 1 is inversely scored. The minimum and maximum possible scores are 20 and 80, respectively. The test–retest method was employed to evaluate the reliability of the State-Trait Anxiety Inventory^25^. The results demonstrated that the State Anxiety Index exhibited a Cronbach's alpha coefficient of 0.92, with a range of 0.65 to 0.86.

### Progressive muscle relaxation (PMR) technique

The first researcher provided instructions on the PMR technique to the trainee participant and monitored the accuracy and duration of each exercise. The second researcher was responsible for regulating the PMR technique on the injured hip, which was undergoing fracture surgery. This approach ensured that no additional injury was incurred.

The PMR technique was implemented according other studies^12,17,26,27^. The technique was initiated for the participants in the intervention group on the night following surgery. The PMR technique was performed three consecutive nights, with each session taking place at night, approximately three hours after the dinner meal, at 9:00 PM. The duration of each session was approximately 30 min. If a mistake was identified in terms of the method or duration of the PMR technique, the first researcher requested that the trainee participant repeat the exercise in the correct method and duration.

In the preliminary stage of the technique, the room environment was tranquilized and environmental stimuli were minimized. The trainee participant was instructed to visit the bathroom and empty their bladder, to wear loose clothing and remove their socks and shoes, to avoid eating a heavy meal before the program, to lie on their back in a comfortable position, to minimize the use of their five senses and to close their eyes. In the implementation phase, the trainee was instructed to contract each of the muscles of the upper limbs, shoulders, head and neck, chest, abdomen, and finally the lower limbs for 10–15 s, followed by a 15–20 s relaxation period. During the course of the exercises, the participant was instructed to take a deep breath through the nose and exhale through the mouth. If the participant begins with the upper left limb and then proceeds to the right limb, this principle can be applied to subsequent movements. Notably, participants in the CG were not administered the PMR technique.

### Data analysis

Data was analyzed in two forms, descriptive and analytical. Descriptive statistics were reported as mean and standard deviation for continuous variables and frequency (percent) for categorical variables. Normality distribution was checked using the Shapiro–Wilk test and histogram charts. The demographic characteristics of the groups underwent comparison using the Chi-square test, Fisher's exact test, and Independent sample t-test. The paired t-test was employed to examine the within-group differences in POP, SQ, and anxiety scores between baseline and one-month follow-up. The Independent t-test was used for the between-group comparison of these scores at baseline, while analysis of covariance (ANCOVA) was used for the one-month follow-up, adjusting for baseline as a covariate. In a linear mixed model, we examined the absolute changes in POP, SQ, and anxiety after interventions, while considering demographic characteristics as a confounding factor. The statistical analysis utilized SPSS version 21.

### Ethics approval

All procedures performed in studies involving human participants were in accordance with the ethical standards of the institutional and/or national research committee and with the 1964 Helsinki declaration and its later amendments or comparable ethical standards (code of ethics IR.SBMU.RETECH.REC.1401.724).

### Consent to participate

Informed consent was obtained from all individual participants included in the study.

## Results

### Baseline characteristics of participants

Table 1 presents the demographic information of the participants. A total of 100 participants took part in the study, with 49 being female and 51 males. The mean values of age in the intervention and control groups were 53.78 ± 6.35 and 53.28 ± 6.66 respectively. Most participants in both groups were married (66%). In the intervention group, 52% were in the diploma and lower education level, compared to 64% in the control group. 38% of the intervention group were homemakers and 40% of the control group were self-employed. The father's role in the family was held by 36% of the intervention group and 34% of the control group. Health insurance coverage was reported by 72% of the intervention group and 56% of the control group. Insufficient income was prevalent among most participants in both groups (74% vs. 66%). Age, gender, marital status, education level, occupation, first role in the family, family members, health insurance, and sufficient income did not differ significantly between the two groups.

**Table 1:** Comparison of demographic variables between PMRG and CG.

Variable	Group		*P* value
	PMRG (n = 50)	CG (n = 50)	
	n (%)	n (%)	
Gender			
Male	26 (52)	25 (50)	0.99^b^
Female	24 (48)	25 (50)	
Marital status			
Single	6 (12)	4 (8)	0.52^c^
Married	33 (66)	33 (66)	
Widow	10 (20)	9 (18)	
Divorced	1(2)	4 (8)	
Education level			
Illiterate and elementary	20 (40)	11(22)	0.13^c^
Diploma and lower	26 (52)	32 (64)	
University	4 (8)	7 (14)	
Occupation			
Homemaker	19 (38)	17 (34)	0.63^c^
Self-employed	14 (28)	20 (40)	
Employee	3 (6)	2 (4)	
Retired	14 (28)	11(22)	
Initial role within the family			
Grandfather	6 (12)	4 (8)	0.19^c^
Grandmother	16 (32)	9 (18)	
Father	18 (36)	17 (34)	
Mother	7 (14)	16 (32)	
Child	3 (6)	4 (8)	
Number of family members			
1	10 (20)	11 (22)	0.83^c^
2	10 (20)	14 (28)	
3	9 (18)	9 (18)	
4	12 (24)	10 (20)	
5 and more	9 (18)	6 (12)	
Mechanism of injury			
Fall from standing height	38 (76)	37 (74)	0.72^c^
Road traffic accident	2 (4)	4 (8)	
Falling from a height of more than 1 m	2 (4)	1 (2)	
Falling from a height of less than 1 m	8 (16)	8 (16)	

Data are presented as Mean ± standard deviation and n (%);

^a^Independent sample t-test.

^b^Fisher’s exact test.

^c^Chi-square test.

*PMRG*, progressive muscle relaxation group; *CG*, control group.

### Within and between-group changes in POP, SQ, and anxiety

The results of within and between-group changes are displayed in Table 2. The paired t-test revealed significant within-group changes in the scores of POP, SQ, and anxiety in both groups (*P* < 0.001). The intervention group experienced a decrease in POP score from 7.50 ± 1.68 to 5.94 ± 1.36, a decrease in SQ score from 13.30 ± 2.86 to 7.04 ± 2.57, and a decrease in anxiety from 57 ± 11.06 to 45.40 ± 8.36 (*P* < 0.001). In terms of between-group changes, the independent sample t-test showed no significant difference between the mean values of POP and anxiety at baseline. Nevertheless, the mean value of SQ in the intervention group was significantly higher than the control group at baseline. ANCOVA test showed that there were significant between-group changes in POP, SQ, and anxiety scores at one-month follow-up (follow-up) with an adjusted baseline as a covariate (*P* < 0.001).

**Table 2:** Comparison of changes in the mean scores of POP, SQ, and anxiety of intervention and control groups.

Variable	Group		*P* value^a^
	PMRG (n = 50)	CG( n = 50)	
POP			
Baseline	7.50 ± 1.68	7.38 ± 1.35	0.69
Follow-up	5.94 ± 1.36	6.56 ± 1.15	0.003*
*P* value^b^	< 0.001***	< 0.001***	
SQ			
Baseline	13.30 ± 2.86	9.92 ± 3.77	< 0.001***
Follow-up	7.04 ± 2.57	8.38 ± 3.50	< 0.001***
* P* value^b^	< 0.001***	< 0.001***	
Anxiety			
Baseline	57 ± 11.06	60.38 ± 8.73	0.09
Follow-up	45.40 ± 8.36	56.12 ± 8.40	< 0.001***
* P* value^b^	< 0.001***	< 0.001***	

Data are presented as Mean ± standard deviation.

^a^*P* values derived from an Independent sample t-test (comparison of mean values between intervention and control group at baseline) and ANCOVA test (comparison of mean values between intervention and control group at follow-up with adjustment of follow-up).

^b^*P* values derived from paired t-test.

*P value ≤ 0.05; ***P value ≤ 0.001.

*POP*, postoperative pain; *SQ*, sleep quality, *PMRG*, progressive muscle relaxation group; *CG*, control group.

### Absolute changes in POP, SQ, and anxiety

Table 3 demonstrates the results of the linear mixed model. The linear mixed model assessed the absolute changes of a dependent variable (POP, SQ, and anxiety) from baseline to follow-up measurements by including group (intervention/ control) and time (baseline/follow-up) as independent factors, and demographic variables as confounders.

**Table 3:** Results of Mixed model with intervention.

Dependent	Independent	B	SE	95%CI^a^	*P* value
POP	Group				
	CG (ref)				
	PMRG	− 0.25	0.19	(− 0.64, 0.14)	0.21
	Time				
	Baseline (ref)				
	Follow-up	− 1.19	0.19	(− 1.57, − 0.80)	< 0.001***
	Group				
SQ	CG (ref)				
	PMRG	1.02	0.48	(0.08, 1.96)	0.03*
	Time				
	Baseline (ref)				
	Follow-up	− 3.9	0.50	(− 4.48, − 2.95)	< 0.001***
	Group				
Anxiety	CG (ref)				
	PMRG	− 7.06	1.31	(− 9.64, − 4.48)	< 0.001***
	Time				
	Baseline (ref)				
	Follow-up	− 7.94	1.32	(− 10.52, − 5.36)	< 0.001***

T_1_ = Baseline measurement; T_2_ = Follow-up (one month later).

Dependent Variable = change from baseline to follow-up in pain, sleep quality, and anxiety.

Demographic variables (age, gender, marital status, education level, and occupation, the first role in the family, family members, health insurance, and sufficient income) were controlled in the model.

^a^95% confidence intervals.

*P value ≤ 0.05; ***P value ≤ 0.001.

*Ref*, references category; *POP*, postoperative pain; *SQ*, sleep quality; *PMRG*, progressive muscle relaxation group; *CG*, control group.

The results showed that the absolute change in POP in the intervention group compared to the control group was not significant (B = − 0.25, *P* = 0.21, 95% CI − 0.64 to 0.14). However, the absolute change of follow-up was 1.19 units significantly lower than the baseline measurement (B = − 1.19, *P* < 0.001, 95% CI − 1.57 to − 0.80).

The absolute change of SQ score in the intervention group was 1.02 units significantly greater than the control group (B = 1.02, *P* < 0.05, 95% CI − 0.08 to 1.96). However, the absolute change of follow-up was 3.9 units significantly lower than the baseline measurement (B = − 3.9, *P* < 0.001, 95% CI − 4.48 to − 2.95).

For the anxiety score, the absolute change in the intervention group was 7.06 units significantly lower than the control group (B = − 7.06, *P* < 0.001, 95% CI − 9.64 to − 4.48). Meanwhile, the absolute change of follow-up was 7.94 units significantly lower than the baseline measurement (B = − 7.94, *P* < 0.001, 95% CI − 10.52 to − 5.36). It is important to note that no specific harm resulting from the intervention was observed during the study.

## Discussion

All three of the study's hypotheses were supported by the findings, which demonstrated that using the PMR technique can help HFPs' POP, SQ, and anxiety.

The first hypothesis, namely that the PMR technique can be employed to reduce POP levels, has been corroborated. The findings of Anshasi et al.^13^, are in accordance with the results of this study, which demonstrate that the PMR technique can significantly reduce the intensity of cancer-related pain in patients receiving palliative care, both immediately and one month after the intervention. The findings of another study indicated that the PMR technique is an effective method for alleviating pain and abdominal distension in patients who have undergone colonoscopy^28^.

In their study, Chen et al.^14^ demonstrated that PMR significantly reduced POP in patients with lumbar disc herniation. Eymir et al.^12^ showed that the utilization of PMR and conventional physiotherapy during the hospitalization period and following discharge of patients undergoing total knee arthroplasty resulted in superior outcomes in terms of pain relief compared to conventional physiotherapy. In their study conducted in Iran, Nazari et al.^29^ demonstrated that PMR is an effective intervention for reducing pain in patients undergoing heart valve replacement surgery. Given the consistency between the results of this study and those of previous studies in this area (all of which indicate that the PMR technique has short-term pain-relieving efficacy), it can be recommended as a relatively simple, non-pharmacological method for patients with hip fractures, with the understanding that further studies are needed to confirm these findings.

The findings pertaining to the second hypothesis of the study indicated that the PMR technique can enhance SQ of HFP. In line with this study finding, the researchers of a study found that the PMR technique has been demonstrated to reduce the severity of symptoms in pregnant women with restless legs syndrome, thereby improving their quality of sleep^30^. Loh et al.^15^ found that PMR significantly reduced sleep disturbances and levels of fatigue, anxiety, and depression compared to the control group over time. In a separate study, the findings indicated that PMR is beneficial in enhancing the SQ of patients undergoing heart valve replacement surgery^29^. Another study demonstrated that PMR can prevent the SQ reduction experienced by patients with pulmonary resection^27^. A study conducted by a group of researchers demonstrated that PMR can be recommended as a means of improving SQ and alleviating fatigue in patients suffering from rheumatoid arthritis^31^. The findings of another study indicated that a four-week program of PMR and BR can be an effective approach to enhancing overall SQ in patients who have undergone CABG^17^. The findings of the present study indicate that a clinically significant improvement in the global SQ can be achieved through the implementation of the PMR technique. Consequently, this approach may be employed as a potential therapeutic option in HFPs' SQ.

The results of the study confirmed the third hypothesis of the study and indicated that the PMR technique can effectively alleviate anxiety of HFP. In accordance with the findings of this study, Nguyen et al. demonstrated that musical intervention with gradual muscle relaxation is a viable approach for reducing anxiety, depression, and stress levels in cancer patients undergoing chemotherapy^19^. Loh et al. found that that PMR is an effective intervention for reducing anxiety and depression in patients with head and neck cancer over time^15^. A study was conducted with the objective of determining the impact of PMR and laughter therapy on women undergoing IVF treatment. The findings indicated that the utilization of the aforementioned techniques resulted in a reduction in trait anxiety with a small effect size and depression with a large effect size on the day of oocyte pick-up^32^. In a study, Zendehdel et al. demonstrated that PMR is an efficacious method for alleviating anxiety associated with the Covid-19 pandemic in pregnant women^33^. The findings of this research demonstrated the efficacy of the PMR technique in reducing anxiety in individuals with hip fractures. Given that studies have indicated that pain and anxiety are interrelate^34^, it can be postulated that implementing this technique while concurrently reducing anxiety may result in reduced pain in patients.

## Conclusion

The study's findings indicated that the PMR technique had a beneficial effect on HFPs' outcomes in the short term, including POP, SQ, and anxiety. From the perspective of beneficial effects on postoperative outcomes, this technique, administered in three sessions during the patient's hospital stay, can be included in the healthcare of HFPs to improve health outcomes.

The main limitations of the study pertain to the older adults' comprehension of their psychological and physical conditions in response to PMR technique, as well as the potential influence of personal, psychological, and cultural differences on the outcomes. The authors of the study contend that it is valuable and unique in that it examined the PMR technique as a non-pharmacological, safe and low-cost method on important health outcomes. Furthermore, the results of the study provided important suggestions for clinical practice.

It is recommended that healthcare providers integrate the PMR technique into the care plan of HFPs with the aim of reducing POP, improving SQ, and reducing anxiety. Nevertheless, further studies are required to substantiate the findings and provide more robust evidence for the efficacy of this intervention. Furthermore, qualitative studies are recommended to investigate the challenges of implementing these interventions in clinical practice from the perspectives of healthcare providers and HFPs. The findings of the study conducted in Iran are applicable to HFPs in other countries.

## Data Availability

The data that support the findings of this study are not openly available due to reasons of sensitivity and are available from the corresponding author upon reasonable request.

## Abbreviations

HF: Hip fractures
HFPs: Hip fractures patients
SQ: Sleep quality
POP: Post-operative pain
PMR: Progressive muscle relaxation
PMRG: Progressive muscle relaxation group
CG: Control group
